# Effectiveness of cardioprotective medication in women with suspected ischemic heart disease syndrome: the NHLBI-sponsored women's ischemia synrome evaluation (WISE) study

**DOI:** 10.1186/1532-429X-13-S1-P169

**Published:** 2011-02-02

**Authors:** Mark Doyle, Gerald Pohost, Lesliee Shaw, Sheryl Kelsey, Diane Vido, Delia Johnson, William J Rogers, William J Rogers, Geetha Rayarao, Barry Sharaf, Carl J Pepine, C Noel Bairey Merz, Robert WW Biederman

**Affiliations:** 1 Allegeny General Hospital, Pittsburgh, PA, USA; 2 Brown University, Providence, RI, USA; 3 Cedars-Sinai Medical Center, Los Angeles, CA, USA; 4 University of Pittsburgh, Pittsburgh, PA, USA; 5 University of Alabama, Birmingham, AL, USA; 6 University of Florida, Gainesville, FL, USA

## Introduction

Knowledge of the effectiveness of cardioprotective medication in women with suspected ischemia is hampered by limited clinical trial data and heterogeneous risk. We assessed the effectiveness of cardioprotective medical treatment in women while adjusting for disease severity determined by cardiovascular magnetic resonance imaging (CMRI).

## Hypothesis

Effectiveness of cardioprotective medication will be evident in women at elevated risk determined by CMRI.

## Methods

Women (n=113), mean age 58±12 years, undergoing coronary angiography for symptoms suggestive of myocardial ischemia additionally underwent myocardial perfusion and cardiac function CMRI evaluation. Previously we identified four ischemic heart disease components associated with adverse cardiac events: 1) coronary artery stenosis > 50%, 2) low global myocardial perfusion, 3) high cardiac energy utilization, and 4) myocardial wall thickness > 10 mm. The latter three disease components were measured using CMRI. Women were stratified into 5 groups based on number of ischemic heart disease components (0-4). Self-reported medication use included angiotensin converting enzyme inhibitors, beta blockers (BB), calcium channel antagonists (CCA) and nitrates. During follow-up (32±17 months) time to first adverse event (death, myocardial infarction, hospitalization for either congestive heart failure or for worsening anginal symptoms) was analyzed. Cox proportional hazard regression was performed to examine the cardioprotective effectiveness of medications when accounting for ischemic heart disease severity.

## Results

Medication usage did not differ across ischemic heart disease strata. Ischemic heart disease stratification predicted adverse cardiac events (hazard ratio 2.1, 95% confidence interval 1.5-2.9, p<0.001) and the medication combination of a BB or CCA and no nitrate were independently associated with reduced risk of events (hazard ratio 0.35, 95% confidence interval 0.14-0.86, p<0.05). The model remained significant after adjustment for age and Framingham risk score.

## Conclusions

Using a combined model of ischemic heart disease severity and medication use, the medication combination of beta-blocker or calcium antagonist and no nitrate were associated with a decreased risk of adverse cardiac events in women. Further investigation of these results should be done with a prospective randomized clinical trial.

